# MRI Findings in a Patient with Known SCAR-16 Type STUB1 Associated Cerebellar Ataxia

**DOI:** 10.5334/jbsr.2902

**Published:** 2022-12-14

**Authors:** Alexander Thorvaldsson, Pir Abdul Ahad Aziz Qureshi, Vikram rao Bollineni

**Affiliations:** 1 The National University Hospital of Iceland, IS; 2 Universitair Ziekenhuis Brussel, BE

**Keywords:** spinocerebellar ataxias, cerebellar ataxia, magnetic resonance imaging, STUB1

## Abstract

Our case report describes a 34-year-old patient sent for magnetic resonance imaging (MRI) after four years of slow onset neurological symptoms. An MRI of her brain showed moderate to severe atrophy of the cerebellum and brainstem. She has a family history of spinocerebellar ataxia and has known STUB1 mutation. Imaging features, genetic analysis, and clinical history are in keeping with the SCAR-16 type of STUB1-associated cerebellar ataxia.

**Teaching Point:**

This case report will help the radiologist to familiarize themselves with the CT and MRI features of STUB1-associated cerebellar ataxia and will provide suggestions to further differentiate between the SCAR-16 and SCA-48 types of STUB1-associated cerebellar ataxia.

## Introduction

Autosomal recessive cerebellar ataxias (ARCA) are a heterogeneous group of autosomal-recessive neurological disorders that are characterized by degeneration or abnormal development of the cerebellum and spinal cord [1]. They usually have an early onset, starting before the age of 40, with peak incidence between ages 30–40 [2]. ARCAs are further defined by their underlying gene mutation, with almost 200 known affected genes [3]. In addition, there are more than 40 genetic types of autosomal dominant cerebellar ataxias (SCAs) [4].

## Case History

**Figure 1:**
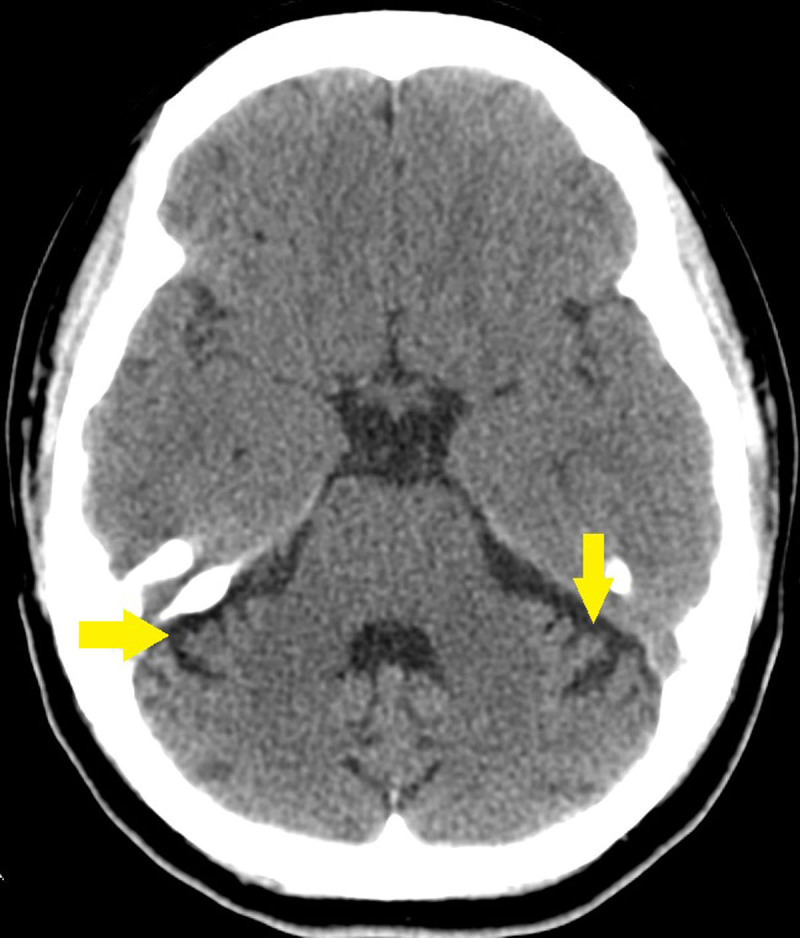
Unenhanced CT axial image showing atrophy of the cerebellar folia (yellow arrows). Note the normal appearance of pons.

A 34-year-old patient with a known family history of spinocerebellar ataxia and known mutation of the STUB1 gene presents to the neurological outpatient clinic with four-year slow onset history of dizziness, slurred speech, and hearing impairment and ataxia. A baseline unenhanced CT examination of the patient’s brain taken 15 years prior showed prominent cerebellar sulci for her age suggestive of marked cerebellar atrophy (Figure 1). Recent magnetic resonance imaging (MRI) of her brain was then performed that showed severe progressive atrophy of the cerebellum with a secondary increase in CSF space in the posterior fossa and relative sparing of the cerebellar tonsils. There was also mild to moderate atrophy of the brainstem, most prominently in the pons and medulla. A smooth notch of about 3.5 mm is also seen in the posterior part of the body of the corpus callosum. No abnormal signal in the brainstem or cerebellum is seen (Figures 2A, B). Further, MRI of the spinal cord showed no abnormalities. Based on the clinical history, radiological findings and genetic analysis, a diagnosis of STUB1-associated cerebellar ataxia SCAR-16 type was made.

**Figure 2A:**
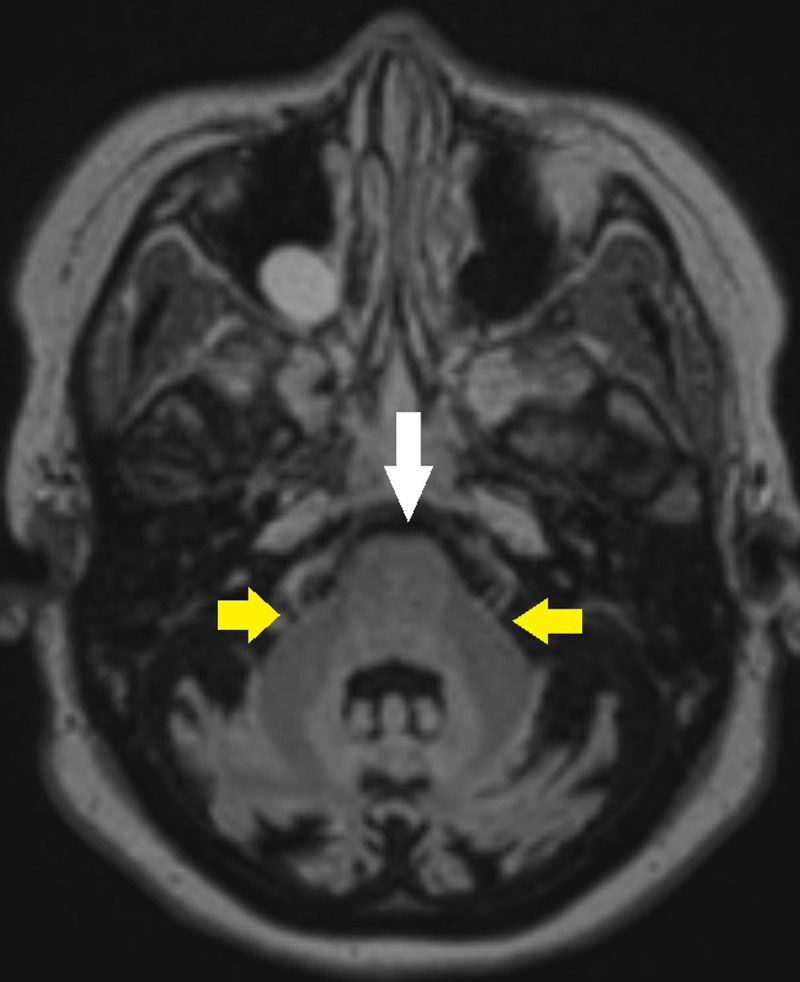
MRI T2 FLAIR axial image showing atrophy of the pons (white arrow) and relatively preserved middle cerebellar peduncles (yellow arrows).

**Figure 2B:**
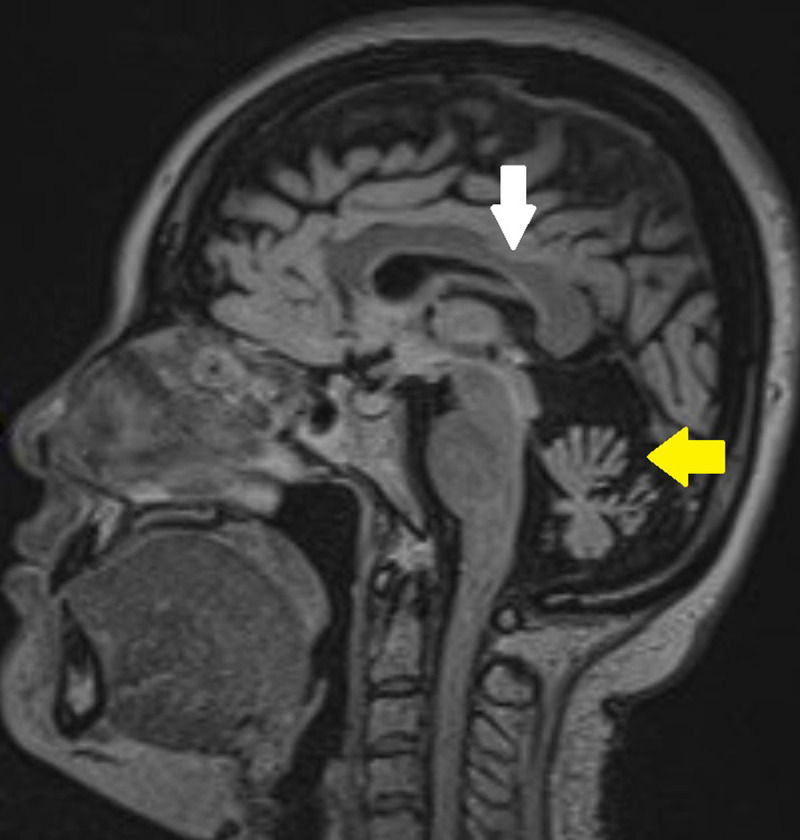
T2 FLAIR sagittal images showing atrophy of cerebellar hemispheres and the vermis (yellow arrows) with notching in the posterior part of body of corpus callosum (white arrow).

## Comments

STUB1 encodes the C-terminus of HSC70-interacting protein (CHIP) that functions as an E3 ubiquitin ligase and co-chaperone. Mutations in the STUB1 gene have been identified to cause autosomal recessive spinocerebellar ataxia type 16 (SCAR-16) as well as a cause for autosomal dominant spinocerebellar ataxia type 48 (SCA-48) [5]. The global prevalence of spinocerebellar ataxia is estimated to be 1–5:10^5^, with the highest rates reported in Portugal, Japan and Southeast Norway. SCAR-16 has a relatively young onset (younger than 30) and SCAR-48 has later onset (20–55 years of age) [2]. It has been proposed to group these diseases by fundamental pathophysiological mechanisms and pathways rather than underlying genetic variants as this approach moves us further towards targeted treatment [3]. MRI helps visualize the main sites of atrophy and signal changes. Imaging features of patients with SCAR-16 phenotype are cerebellar atrophy associated with atrophy of the pons, midbrain, or cerebral cortex and thinning of the corpus callosum. Imaging features of SCA-48 are cerebellar atrophy that is more pronounced in the posterior areas, mild cortical atrophy, and sometimes T2 hyperintensity affecting the dentate nucleus extending to the middle cerebellar peduncles; this feature is not present in the SCAR-16 type [6]. The image findings in our patient showed characteristic progressive atrophy of the cerebellum, which is consistent with the phenotypic characteristic of both SCAR-16 and SCA-48 types of STUB1-associated cerebellar ataxia. However, the addition of atrophy of the brainstem and the lack of T2 signal changes in the dentate nucleus is more in keeping with imaging features of SCAR-16. It should be noted, however, that the demonstrated findings may not be very specific for the STUB1-mutation itself as these findings may also demonstrated in other spinocerebellar ataxia syndromes and related disorders, but when the mutation is confirmed, imaging may help in determining the subtype.

## Conclusion

In conclusion, our case report presents the radiological features of the SCAR-16 type STUB1 associated cerebellar ataxia, which is a rare genetic disorder and provides suggestions for the reporting radiologists to help differentiate between the SCAR-16 and SCA-48 types of STUB1 associated cerebellar ataxia.
